# Patient Education in Bariatric Surgery: Can Artificial Intelligence–Based Chatbots Bridge the Knowledge Gap?

**DOI:** 10.1155/jobe/2376530

**Published:** 2026-02-12

**Authors:** Amirreza Izadi, Hesam Mosavari, Ali Hosseininasab, Ali Jaliliyan, Arzhang Jafari, Mohammadhosein Akhlaghpasand, Aghil Rostami, Maziar Moradi-Lakeh, Foolad Eghbali

**Affiliations:** ^1^ Department of Surgery, Surgery Research Center, School of Medicine, Rasool-E Akram Hospital, Iran University of Medical Sciences, Tehran, Iran, iums.ac.ir; ^2^ Artificial Intelligence in Health Research Center, Iran University of Medical Sciences, Tehran, Iran, iums.ac.ir; ^3^ Functional Neurosurgery Research Center, Shohada Tajrish Comprehensive Neurosurgical Center of Excellence, Shahid Beheshti University of Medical Sciences, Tehran, Iran, sbmu.ac.ir; ^4^ Department of Epidemiology and Biostatistics, School of Public Health, Tehran University of Medical Sciences, Tehran, Iran, tums.ac.ir; ^5^ Gastrointestinal and Liver Diseases Research Center, Iran University of Medical Sciences, Tehran, Iran, iums.ac.ir; ^6^ Division of Minimally Invasive and Bariatric Surgery, Department of Surgery, Minimally Invasive Surgery Research Center, Rasool-E Akram Hospital, School of Medicine, Iran University of Medical Sciences, Tehran, Iran, iums.ac.ir

**Keywords:** AI chatbots, bariatric surgery, ChatGPT, healthcare technology, laparoscopic sleeve gastrectomy, patient education

## Abstract

**Background:**

The global obesity epidemic challenges health systems, driving people to seek metabolic and bariatric surgery (MBS), especially laparoscopic sleeve gastrectomy (LSG). Many MBS centers have limited resources for patient education, creating knowledge gaps that lead patients to search online. AI chatbots, such as ChatGPT, can provide reliable medical information, though concerns about accuracy and completeness remain.

**Methods:**

The study involved four fellowship‐trained minimally invasive surgeons (MISs), nine fellows (MIFs), and two general practitioners (GPs) in the MBS multidisciplinary team from March 1, 2024, to March 30, 2024. Seven AI chatbots were selected, including ChatGPT 3.5 and 4, Bard, Bing, Claude, Llama, and Perplexity, based on their public availability on December 1, 2023. Forty patient questions regarding LSG were sourced from social media, MBS organizations, and online forums. Experts and chatbots answered these questions, with their responses evaluated for accuracy and comprehensiveness on a 5‐point scale. Statistical analyses compared groups’ performance.

**Results:**

Chatbots demonstrated a higher overall performance score (2.55 ± 0.95) compared to the expert group (1.92 ± 1.32, *p* < 0.001). Among chatbots, ChatGPT‐4 achieved the highest performance (2.94 ± 0.24), while Llama had the lowest (2.15 ± 1.23). Expert group scores were highest for MISs (2.36 ± 1.09), followed by GPs (1.90 ± 1.36) and MIFs (1.75 ± 1.36). The readability of chatbot responses was assessed using Flesch–Kincaid scores, revealing that most responses required reading levels between the 11th grade and college level. Furthermore, chatbots exhibited fair reliability and reproducibility in response consistency, with ChatGPT‐4 showing the highest test–retest reliability.

**Conclusion:**

AI chatbots generated accurate and comprehensive answers to common bariatric patient questions, suggesting promise as a scalable aid for patient education. However, readability often exceeds recommended levels, performance varies by model, occasional inaccuracies occur, and medicolegal considerations remain unresolved. Accordingly, chatbots should complement clinician counseling, and future work should improve readability and reliability and evaluate real‐world safety and impact.

## 1. Introduction

### 1.1. Obesity and Bariatric Surgery

The obesity epidemic poses a significant global health challenge; more than 1 billion people worldwide suffer from obesity, with a rising trend. WHO estimates that by 2025, approximately 167 million people will become less healthy because of obesity and obesity‐related diseases [1]. Obesity is a risk factor for a variety of conditions, for instance, diabetes, cardiovascular disease, cancer, nonalcoholic fatty liver disease, cirrhosis, and liver failure [2]. It contributes substantially to morbidity and mortality rates [3], leading to increased healthcare expenditures and strain on human resources within the healthcare system, affecting those directly impacted and society [4–6].

Metabolic and bariatric surgery (MBS) has emerged as a solution, offering substantial and durable weight loss [7, 8]. Among different approaches in MBS, laparoscopic sleeve gastrectomy (LSG) is a fairly safe weight loss surgical procedure that represents 46% of all bariatric surgeries worldwide [9, 10], with a significant positive impact on improving obesity comorbidities such as hypertension, type 2 diabetes, dyslipidemias, and obstructive sleep apnea [10, 11].

### 1.2. Patient Education and Online Resources

Patients frequently seek to comprehend and gain insight into their medical conditions and treatment options. Several studies have underscored the significance of patient education, particularly in chronic diseases [12]. While MBS represents a singular operative intervention, multidisciplinary and regular postoperative care in MBS patients is consistent with the model of care for chronic disease. Patient education improves the degree of weight loss achieved regardless of the procedure [13]. It also plays a crucial role in adherence to lifestyle modifications and improves compliance and weight loss outcomes following MBS [14, 15]. Sufficient education can empower patients to make informed decisions, reduce anxiety, and actively engage in treatment deliberations, aiding them in comprehending various facets of the proposed care plan and ultimately refining the course of recovery and surgical outcomes [12, 15, 16].

Traditionally, patients have sought medical information from physicians, but inherent limitations constrain this avenue, especially in access to physicians and its costs. Hence, patients increasingly turn to online sources for information about their conditions, especially when they feel healthcare providers fail to answer [17, 18]. The ambiguity inherent in acquiring knowledge and obtaining information from the Internet is highlighted by the fact that various search engines produce significantly different results for identical search queries, and slight variations in wording can lead to significantly divergent outcomes. Consequently, it becomes challenging to anticipate what users will encounter during their searches. Furthermore, ranking a website in search results is complex, relying on several factors, such as popularity and inbound links. This process is subject to manipulation, and a site’s quality does not always match its ranking or search visibility [19, 20]. This algorithm makes health information vulnerable to marketing, propaganda, and spreading false information. There is also heterogeneity in the reliability and readability of health‐related content for the general population [21, 22].

### 1.3. Large Language Models (LLMs) for Patient Education

In response to this complex set of challenges, the potential exhibited by chatbots could be utilized to provide patients with sufficient and accurate information. LLMs have recently become publicly available through conversational AI models called “Chatbots.” These models are trained using various online resources, including books and articles [23]. This training enables chatbots to comprehend the intricacies of users’ inquiries and adeptly address a wide range of user queries, including those related to medical concerns [24].

Previous studies have highlighted the clinically valid insights of AI‐powered chatbots [25, 26]. Despite their promising performance, numerous studies have also highlighted risks and weaknesses—such as reference inaccuracies, uncertain provenance and quality of training datasets, and AI hallucinations—prompting warnings against using these systems for medical information and research [27–30]. Furthermore, as an example, ChatGPT and GPT‐4 were trained on data only until 2021 and generally lack access to information hidden behind paywalls [23]. Due to the proprietary nature of their training, it is challenging to anticipate inherent model biases and errors in advance [31].

Integrating AI in this delicate domain necessitates carefully examining its capabilities, reliability, risks, and benefits for patients’ health and well‐being. This study aims to critically evaluate the performance of chatbots in providing adequate and accurate answers to patient questions on LSG, comparing their responses to those of bariatric surgery experts as the conventional way of ascertaining medical and health information. By doing so, it seeks to shed light on the chatbot’s strengths, limitations, and potential to complement conventional patient education methods.

## 2. Materials and Methods

### 2.1. Study Design

This observational study compared the performance of chatbots and bariatric surgery experts at the Center of Minimally Invasive and Bariatric Surgery, Rasool‐E Akram Hospital, affiliated with the Iran University of Medical Sciences, in answering patients’ frequently asked questions (FAQs) about LSG. It was conducted using the principles outlined in the Declaration of Helsinki and received approval from the Institutional Review Board (IRB) under the ethics code IR.IUMS.REC.1402.982. Informed written consent was obtained from all the human participants of this study.

### 2.2. Participant

Four fellowship‐trained, board‐certified minimally invasive surgeons (MISs), nine minimally invasive surgery fellows (MIFs), and two general practitioners (GPs) participated in the study from March 1, 2024, to March 30, 2024. For MISs, inclusion required active practice and completing over 50 LSG surgeries in the previous year. The authors of this study were excluded. MIFs were included if they had completed at least six months of training and had performed over 50 LSG surgeries. GPs (specially trained and responsible for initial patient consultation and guiding them throughout getting approved for MBS) were included if they had more than 1 year of experience. We will use the terms “expert group” and “bariatric surgery experts,” referring to all MISs, MIFs, and GPs.

### 2.3. Chatbot Selection

We selected seven publicly available chatbots: ChatGPT versions 3.5 and 4 (dated October 30, 2023), Bard (dated October 30, 2023), Bing (latest version as of December 1, 2023), Anthropic’s Claude (version 2.0 dated December 1, 2023), Llama (version 2b7, dated December 1, 2023), and Perplexity (latest version as of December 1, 2023). We chose chatbots based on their public availability and general popularity. We excluded chatbots designed for narrow, specialized purposes to focus the analysis on versatile, general‐purpose conversational agents.

### 2.4. Question Selection

We created the question set by gathering input from several sources that patients commonly access to learn about bariatric surgery. We retrieved questions from social media platforms (e.g., Twitter, Instagram) and the FAQ section of leading organizations in MBS like the International Federation for the Surgery of Obesity and Metabolic Disorders (IFSO), the American Society for Metabolic and Bariatric Surgery (ASMBS), Society of American Gastrointestinal and Endoscopic Surgeons (SAGES), and the Iran Society for Metabolic and Bariatric Surgery (ISMBS) websites. Additionally, we reviewed online patient forums and education materials to find common patient questions.

Through this process, we compiled an initial pool of 200 questions on all aspects of LSG. After removing duplicate, unclear, too general, overly specialized, and ambiguous questions, we narrowed the list to 80 viable questions for the question set. We categorized 80 questions into five topics, each with at least 10 questions: outcome and expectation, preoperative care, recovery and postoperative care, risks and complications, and lifestyle modifications.

A team of three fellowship‐trained, board‐certified MISs reviewed the final pool of 80 questions to identify and eliminate any ambiguities. By refining together and focusing on clarity while maintaining the original objectives and topics covered, we arrived at 40 clear, focused, patient‐centered questions for LSG surgery. We also considered accessibility by trying to avoid complex medical terminology and encouraging patient empowerment through the style and content of the questions. The English version of all Questions is available in the appendix (Supporting Table A1).

Questions originally in English were translated into Persian using a standard forward–backward translation process, which included a cognitive interview with five surgical residents and five bariatric surgery candidates. This focus group offered feedback, which we used to identify and amend ambiguous phrases or sentences to create a final version aligned with patients’ real concerns. Subsequently, two MISs meticulously reviewed the translated questions to preserve the original aims and topics, making minor changes to enhance clarity and readability. Questions sourced from Persian materials underwent a similar translation process into English, facilitated by cognitive interviews with five surgical residents proficient in both languages. Again, two MISs meticulously reviewed the translated questions to preserve the original aims and topics. English questions were used in their original form as they were extracted.

Questions were presented to expert groups in Persian. The answers by expert groups were also in Persian, and the Persian transcripts were examined. Questions were presented to the chatbots in English, and English answers were analyzed.

### 2.5. Answer Sheet

Two MISs compiled the answer sheet, including critical clinical practice recommendations based on MBS guidelines from SAGES, IFSO, and ASMBS [7, 32–34]. If the answer to a question could not be found in the guidelines, we utilized the most recent high‐quality systematic reviews and meta‐analyses and the answers provided in the FAQ section of the ASMBS or IFSO official websites [35, 36]. An independent fellowship‐trained, board‐certified MIS assessed the answer sheet to determine its completeness and comprehensiveness. Feedback on item content, wording, and relevance was integrated to improve the final answer sheet utilized in the present study to compare and evaluate the responses of bariatric surgery experts and chatbots. The English version of the answer sheet is available in the appendix (Supporting Table A1).

### 2.6. Taking the Exam

We invited participants to take an exam at a designated location. Their verbal responses were audio‐recorded. Two surgical interns independently transcribed the recordings word‐for‐word into text documents. One author then reviewed the original audio recordings and final transcripts for accuracy.

Chatbots were presented with questions one at a time on separate tabs to prevent previous answers from influencing responses. Each chatbot was asked the same set of questions twice within the study period to evaluate the consistency and reliability of responses. All responses were unidentified and compiled into spreadsheets for scoring.

### 2.7. Scoring

Two MISs independently rated the accuracy and comprehensiveness of each response based on a scoring sheet on a 5‐point rating scale. The scale was defined as 3 = comprehensive and accurate; 2 = accurate but incomplete; 1 = partially accurate; 0 = inaccurate or missing key information; −1 = completely inaccurate, potentially harmful, or misleading. Any differences in ratings among the two reviewers were resolved through discussion and, if necessary, the involvement of a third reviewer to reach a consensus. A panel of three MISs agreed to score the missing answer and “I don’t know” responses as completely inaccurate and potentially harmful. The final score for each individual chatbot and bariatric surgery expert was calculated by summing the ratings across all questions. Mean and standard deviations (SDs) were used to report the overall performance of each group of bariatric surgery experts (e.g., MISs, MIFs, GPs) as well as the chatbot group. Each chatbot’s mean and SD reflect the average score obtained from two test iterations.

The readability assessment of chatbots’ responses (in English) was done using multiple readability metrics, including Flesch–Kincaid Grade Level (FKGL), Flesch–Kincaid Reading Ease (FRE), Flesch Readability Category (FRC), Flesch School Level (FSL), Gunning Fog Score (FGI), Coleman–Liau Index (CLI), Automated Readability Index Score (ARI‐S), and Automated Readability Index Age (ARI‐A). The readability assessment was performed using the readability library version 0.3.1 (Andreasvc, 2019) and Python version 3.12.1 (Python Software Foundation, 2023).

### 2.8. Statistical Analysis

We conducted all analyses using IBM SPSS Statistics for Windows, Version 27.0 (IBM Corp., 2020). We used descriptive statistics to characterize the distribution of performance scores for each expert group and chatbot. In assessing the normality of the data distribution, we employed Kolmogorov–Smirnov, using an alpha level of 0.05 for significance. We compared the mean accuracy of overall scores and scores for each subcategory between groups using one‐way ANOVA analysis for normally distributed data and the Kruskal–Wallis test for non‐normally distributed data. The analysis was used to assess group performance and identify significant differences in accuracy between MISs, MIFs, GPs, and chatbots.

Additionally, the percentage of irrelevant/harmful responses was compared to assess the critical error difference between groups. We performed a test–retest analysis using the intraclass correlation coefficient (ICC) to assess the repeatability and consistency of chatbot responses over time. A one‐way random effects model with consistency was employed to measure the degree of agreement between the ratings. ICC values were interpreted as follows: values less than 0.40 indicated poor reliability, values between 0.40 and 0.59 indicated fair reliability, values between 0.60 and 0.74 indicated good reliability, and values of 0.75 or higher indicated excellent reliability.

## 3. Results

In this study sample, we compared the performance of bariatric surgery experts and AI‐powered chatbots in answering 40 frequent patients’ questions, covering a range of inquiries including outcome and expectation, preoperative care, recovery and postoperative care, risks and complications, and lifestyle modifications (Table 1).

**TABLE 1:** The score of experts and chatbots for answering common questions about laparoscopic sleeve gastrectomy in total and each category.

	Total	Outcome and expectation	Preoperative care	Recovery and postoperative care	Risks and complications	Lifestyle modifications
MISs^a^	2.36 (±1.09)^d^	2.67 (±0.58)	2.42 (±0.97)	2.50 (±1.21)	1.86 (±1.32)	2.25 (±1.13)
MIFs^b^	1.75 (±1.36)	2.26 (±1.08)	1.42 (±1.46)	1.90 (±1.41)	1.64 (±1.29)	1.38 (±1.39)
GPs^c^	1.90 (±1.36)	2.11 (±1.18)	1.58 (±1.50)	2.33 (±1.24)	1.79 (±1.37)	1.56 (±1.50)
Bing	2.46 (±1.03)	2.50 (±0.98)	1.58 (±1.38)	2.78 (±0.55)	2.29 (±1.33)	2.83 (±0.51)
Bard	2.44(±1.02)	2.94 (±0.23)	2.08 (±1.08)	2.11 (±1.41)	2.57 (±0.65)	2.39 (±1.09)
Claude	2.59 (±0.87)	2.72 (±0.57)	2.08 (±1.08)	2.67 (±0.84)	2.64 (±0.84)	2.67 (±0.97)
Llama	2.15 (±1.23)	2.28 (±1.23)	2.00 (±1.28)	2.17 (±1.34)	2.29 (±0.99)	2.00 (±1.37)
Perplexity	2.36 (±1.15)	2.39 (±1.09)	2.25 (±0.97)	2.50 (±1.15)	2.14 (±1.46)	2.44 (±1.15)
ChatGPT‐3.5	2.91 (±0.33)	3.00	2.58 (±0.51)	3.00	3.00	2.89 (±0.47)
ChatGPT‐4	2.94 (±0.24)	3.00	2.67 (±0.49)	3.00	2.93 (±0.27)	3.00
Expert group	1.92 (±1.32)	2.34 (±1.00)	1.69 (±1.41)	2.10 (±1.36)	1.71 (±1.29)	1.62 (±1.39)
Chatbot group	2.55 (±0.95)	2.62 (±0.87)	2.18 (±1.04)	2.60 (±0.97)	2.55 (±0.95)	2.60 (±0.94)

^a^Minimally invasive surgeons.

^b^Minimally invasive surgery fellows.

^c^General practitioners.

^d^The numbers represent the mean ± standard deviation.

### 3.1. Chatbot Group Performance Versus Expert Group Performance

**FIGURE 1:**
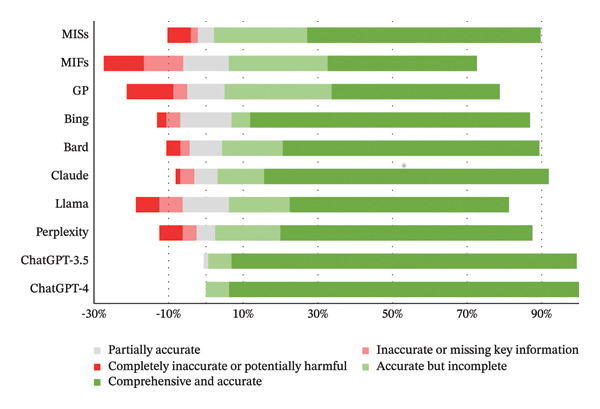
Response accuracy of each group across all questions, categorized by the degree of accuracy and completeness. MISs, minimally invasive surgeons; MIFs, minimally invasive surgery fellows; GPs, general practitioners.

The overall chatbots’ performance score (2.55 ± 0.95) was higher than the overall bariatric surgery experts’ performance score (1.92 ± 1.32), which was statistically significant (*p* < 0.001) (Table 1, Figure 1).

### 3.2. Expert Group Performance

Among the bariatric surgery experts, MISs provided the most accurate and comprehensive answers (2.36 ± 1.09), followed by GPs (1.90 ± 1.36) and MIFs (1.75 ± 1.36) (Table 1, Figure 1). There was a statistically significant difference between MISs and MIFs bariatric surgery experts (*p* = 0.011) (Table 2).

**TABLE 2:** Pairwise comparison *p* values between groups, derived from ANOVA and Tukey’s post hoc tests.

	MISs^a^	MIFs	GPs	Bing	Bard	Claude	Llama	Perplexity	ChatGPT‐3.5
MIFs^b^	0.011^∗^								
GPs^c^	0.507	0.997							
Bing	1.000	0.029^∗^	0.422						
Bard	1.000	0.038^∗^	0.481	1.000					
Claude	0.979	0.007^∗^	0.193	1.000	1.000				
Llama	0.990	0.517	0.987	0.946	0.967	0.729			
Perplexity	1.000	0.084	0.668	1.000	1.000	0.994	0.996		
ChatGPT‐3.5	0.262	< 0.001^∗^	0.014^∗^	0.699	0.637	0.993	0.111	0.452	
ChatGPT‐4	0.216	< 0.001^∗^	0.014^∗^	0.637	0.574	0.900	0.091	0.394	1.000

^a^Minimally invasive surgeons.

^b^Minimally invasive surgery fellows.

^c^General practitioners.

^∗^Statistically significant.

### 3.3. Chatbot Group Performance

The range of performance scores for chatbots was from 2.15 (Llama) to 2.94 (ChatGPT‐4). Chat‐GPT‐4 achieved the highest performance score (2.94 ± 0.24), closely followed by Chat‐GPT‐3.5 (2.91 ± 0.33) (Table 1). There was no statistically significant difference between the performance scores of different chatbots (Table 2).

### 3.4. Top‐Performing Chatbots Versus Expert Groups

The top two chatbots—ChatGPT‐4 and ChatGPT‐3.5—achieved higher performance scores than MISs (the top performer among expert groups) (2.94 ± 0.24, 2.91 ± 0.33 vs. 2.36 ± 1.09, respectively) (Table 1). However, there was no statistically significant difference between them (Table 2). Additionally, there was a statistically significant difference between the other two expert groups (e.g., MIFs, GPs) and the top two chatbots (*p* < 0.05). The pairwise comparison *p* values between different chatbots and expert groups are shown in Table 2.

### 3.5. Worst‐Performing Chatbots Versus Expert Groups

The worst‐performing chatbot, Llama (2.15 ± 1.23), achieved a lower performance score than MISs (2.36 ± 1.09) but higher than other expert groups (Table 1). There was no statistical difference between Llama and any of the expert groups (Table 2).

### 3.6. Domain‐Based Performance

**FIGURE 2:**
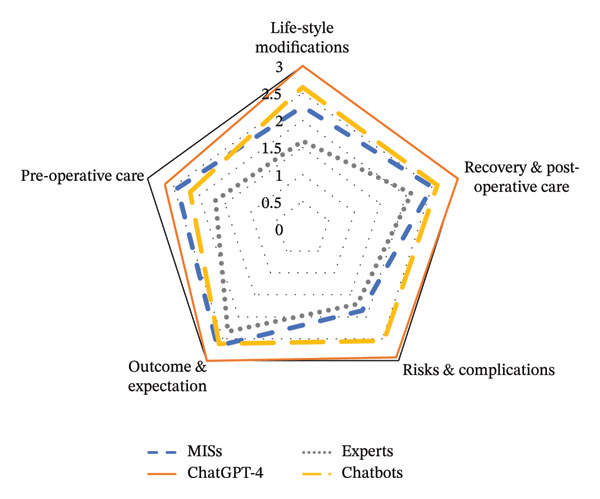
Comparison of performance score between the top‐performing chatbot (ChatGPT‐4), the top‐performing expert group (MISs), all chatbots (Chatbots), and all expert groups (Experts) across various categories. Minimally invasive surgeons, MISs.

In domain‐based analysis, expert groups’ performance scores ranged from 1.62 ± 1.39 (lifestyle modification) to 2.34 ± 1.00 (outcome and expectation) (Table 1, Figure 2). The overall chatbot performance scores were significantly higher than the average scores of expert groups across all categories (*p* < 0.05). MISs achieved a higher performance score among expert groups, but the difference was not statistically significant. ChatGPT‐3.5 and ChatGPT‐4 achieved higher performance than other chatbots, but no statistically significant differences were observed (Table 2). Although the top‐performing chatbots (e.g., ChatGPT‐4, ChatGPT‐3.5) scored higher than MISs, MISs scored higher than the worst‐performing chatbots (Llama) in all categories except for the recovery and postoperative care category. MISs also scored higher than perplexity in outcome and expectation and preoperative care categories, but these differences were not statistically significant (see appendix, Supporting Tables A5 and A6). The only statistically significant differences were evident between MIFs and top‐performing chatbots in recovery and postoperative care, risk and complications, and lifestyle modification categories (see appendix, Supporting Tables A2–A6, and Supporting Figures A1–A10).

### 3.7. Incorrect and Misleading Answers

In this study, responses rated as “0” (inaccurate or missing key information) or “‐1” (completely inaccurate, potentially harmful, or misleading) on our scoring system were considered “incorrect.” A total of 111 (17.34%) responses from experts were incorrect, with 63 (9.84%) responses classified as potentially harmful or misleading. MIFs exhibited a higher proportion of incorrect answers at 21.25% (*n* = 85), with 10.75% (*n* = 43) categorized as potentially harmful or misleading. Most of the wrong or misleading answers belonged to the lifestyle modification category (*n* = 39, 27.08%), and 43.59% (*n* = 17) of them were potentially harmful or misleading.

In contrast, chatbots demonstrated a lower rate of incorrect responses, accounting for 5.36% (*n* = 32) of their total responses, of which 50% (*n* = 16) were marked as potentially harmful or misleading. Among chatbots, Llama had the highest percentage of incorrect answers, 12.5% (*n* = 10), of which 50% (*n* = 5) were potentially harmful or misleading. None of the responses from ChatGPT‐3.5 and ChatGPT‐4 were marked as incorrect.

### 3.8. Readability

**FIGURE 3:**
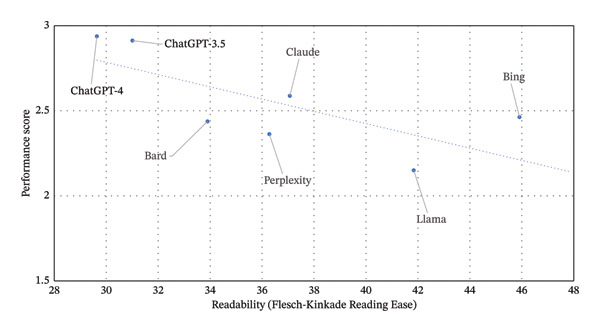
The inverse relationship between performance score and readability (measured by the Flesch–Kinkade Reading Ease) for various chatbots, with a downward trend indicating that higher accuracy correlates with lower readability.

The overall Flesch–Kincaid Reading Ease score for all the chatbots was 36.52 ± 5.79, translating into a range from 11th grade to college level, based on Flesch grade (Table 3). ChatGPT‐3.5 showed the lowest readability, with a reading score of 29.63 associated with “very confusing,” and Bard showed the highest readability, with a reading score of 45.91 associated with “difficult.” Other chatbots scored within the relatively “difficult” range, with reading scores between 31 and 45.91. Further analysis suggests that their language requires the reading skills expected of 11th‐grade to college‐graduate students. Figure 3 shows the Flesch–Kinkade Reading Ease versus the performance scores.

**TABLE 3:** Readability analysis for chatbots.

	Bard	Llama	Claude‐2	Bing	Perplexity	Chatgpt‐4	Chatgp‐3.5	Overall (mean ± sd)
FKGL^a^	11	13	12	11	13	13	13	—
FRE^b^	45.91	33.90	37.07	41.84	36.28	31.01	29.64	36.52 ± 5.79
FRC^c^	Difficult	Difficult	Difficult	Difficult	Difficult	Difficult	Very Difficult	—
FSL^d^	College	College	College	College	College	College	College Graduate	—
FGI^e^	13.89	15.72	15.06	14.22	15.96	15.71	16.65	15.32 ± 0.98
CLI^f^	12.83	14.55	14.63	12.88	12.53	15.25	15.53	14.03 ± 1.25
ARI‐S^g^	11.23	13.15	12.27	10.85	11.80	12.77	13.9	12.28 ± 1.08
ARI‐A^h^	17–18	24+	18–24	16–17	17–18	18–24	24+	—

^a^Flesch–Kincaid Grade Level.

^b^Flesch–Kincaid Reading Ease.

^c^Flesch Readability Category.

^d^Flesch School Level.

^e^Gunning Fog Score.

^f^Coleman–Liau Index.

^g^Automated Readability Index Score.

^h^Automated Readability Index Age.

### 3.9. Reproducibility and Reliability of Responses

The results showed good to poor reliability, with ICC ranging from 0.23 to 0.61. ChatGPT‐3.5 had the highest reproducibility, achieving 95% of the same scores for 40 questions (ICC = 0.586, *p* = 0.003). This was closely followed by ChatGPT‐4 with 92% of identical scores for answers (ICC = 0.541, *p* = 0.008) (Table 4).

**TABLE 4:** Reproducibility and reliability of chatbot responses in rest–retest analysis.

	Identical scores for answers, *n* (%)	ICC^a^	*p* value	Reliability
Bard	20 (50%)	0.212	0.228	Poor
Llama	19 (47.5%)	0.281	0.151	Poor
Claude	25 (62.5%)	0.362	0.081	Poor
Perplexity	25 (62.5%)	0.540	0.008	Fair
Chat‐GPT4	37 (92%)	0.541	0.008	Fair
Bing	25 (62.5%)	0.574	0.004	Fair
Chat‐GPT3.5	38 (95%)	0.586	0.003	Fair

^a^Intraclass correlation coefficient.

## 4. Discussion

### 4.1. Principal Finding

Recently, AI‐based chatbots have gained popularity and are now commonly used for various purposes, including looking for medical and health information [37]. We conducted this study to evaluate chatbots’ educational value and limitations. This study found that AI‐based chatbots are able to provide accurate and comprehensive answers with fair reliability to frequently asked questions about LSG. However, responsible deployment should be tempered by recognition of important constraints—namely, variability in users’ digital and health literacy, proficiency in prompt formulation, and unresolved issues related to safety, security, medicolegal responsibility, and liability.

### 4.2. Accuracy and Comprehensiveness

Differing from previous studies [38–40], we selected questions that chatbots are realistically expected to answer correctly. Chatbots are trained using widely available information from the Internet and do not undergo specialized medical training [41]. They are more suited for providing general information to patients rather than answering complex medical queries. Therefore, we focused on general patient questions that may not have been addressed by surgeons or other members of a bariatric multidisciplinary team. These are typically the questions that patients turn to online resources to find answers to. However, using search engines for this purpose has drawbacks, such as providing biased information for marketing purposes [19].

Chatbots’ responses achieved higher accuracy and completeness scores than those of the expert group, with a higher percentage of accurate and comprehensive answers and a lower rate of misleading and potentially harmful answers. Our findings align with the study conducted by Samaan et al., which showed that ChatGPT‐3.5 responded comprehensively to 131 out of 151 patient questions (86.8%) [38]. Similarly, in our study, 426 out of 560 (76.07%) of chatbots’ responses scored the highest (comprehensive and accurate). A study by Haver et al. found that ChatGPT provided appropriate responses for 88% of common questions related to breast cancer prevention and screening, as evaluated by fellowship‐trained breast radiologists [42]. In a study by Ye et al., rheumatologists and patients evaluated the responses generated by ChatGPT‐4 and rheumatologists using a survey focused on patient questions about rheumatology. Patients’ ratings exhibited a parallel trend, with no statistically significant difference between physician and chatbot‐generated answers. Conversely, rheumatologists rated the physician‐generated responses superior in comprehensiveness and accuracy [39]. This discrepancy in findings may stem from the methodology employed, particularly in selecting and curating questions for the study.

Among experts, MISs scored highest; GPs were numerically above MIFs, but the difference was not significant (Tukey *p* = 0.997). In our center, GPs have greater day‐to‐day experience guiding and educating patients throughout the bariatric journey and serve as the first line for answering patient questions, so their knowledge aligns more closely with our question list. Fellows may focus more on operative and perioperative technical issues; domain‐level trends were similar but underpowered and remain exploratory.

### 4.3. Incorrect Responses and Errors

The best‐performing chatbots (ChatGPT‐4 and ChatGPT‐3.5) did not receive 0 or ‐1 scores for any of their answers (meaning inaccurate or missing key information and completely inaccurate, potentially harmful, or misleading, respectively). However, 32 of 560 (5.71%) chatbots’ answers received either a −1 or 0 score. Discrepancies arise when chatbots encounter questions that demand responses based on heterogeneous online information:

Q: How long until I can start exercising after laparoscopic sleeve gastrectomy? Range of weeks

Bard: Light exercises […] can be resumed within the first week after surgery. Moderate exercises […] can be resumed within the second week after surgery. Strenuous exercises: Running, heavy lifting, and contact sports can be resumed within the fourth week after surgery.

(Accessed dated December 23, 2023)

The issue of LLMs assigning weights to online resources during the learning process needs to be clarified. It lacks clarity, particularly regarding how different resources with varying levels of reliability and quality should be prioritized. There is currently no consensus on the ideal time to begin exercising after bariatric surgery, with protocols ranging from 7 days to 6 months or more postsurgery [43]. The protocol for postoperative exercise in our center prohibits patients from starting strenuous activity and heavy lifting for at least 8 weeks. Additionally, relevant guidelines recommend avoiding lifting weights of more than 15 lbs during the first 6 weeks after surgery [44].

To minimize the risk of AI‐powered chatbot critical errors, educating the public about AI chatbot limitations, such as AI hallucinations and reasoning constraints, is essential to address negative sentiments and promote effective usage techniques [45]. For example, self‐checking (asking the question multiple times) to see if any divergent or contradictions happen could help find and avoid chatbot errors [46]. Also, some chatbots like Bing and Perplexity cite relevant references when responding to queries. Despite their relatively lower performance compared to ChatGPT and Claude in our study, the ability to fact‐check answers against corresponding references provides a valuable opportunity to verify the accuracy and reliability of the information provided.

### 4.4. Readability

Chatbots with better performance tend to produce responses of greater complexity, characterized by lower Flesch Reading Ease Scores falling within the “very confusing” range. Conversely, models such as Bard and Bing (with lower performance scores) tend to generate relatively more easily understandable answers. However, our findings indicate a potential literacy gap, with most chatbots mandating individuals from an 11th‐ to 14th‐grade education level for comprehension by the average patient. Importantly, the American Medical Association and the National Institutes of Health advise that patient‐facing materials be written at a sixth‐ and eighth‐grade reading level, underscoring that the 11th–14th‐grade readability we observed is misaligned with best‐practice standards [47].

Notably, ChatGPT‐4 exhibits enhanced comprehension of questions, adeptly providing only the relevant information requested without unnecessary detail. This sensitivity to phrases such as “yes or no” or “in three sentences” is less pronounced in ChatGPT‐3.5 and Claude. However, they provide sufficient and relevant information as requested by the questions:

Q: Can laparoscopic sleeve gastrectomy lead to long‐term nutritional deficiencies? Yes or No

ChatGPT‐4: Yes.

(Accessed dated December 28, 2023)

Q: Can laparoscopic sleeve gastrectomy lead to long‐term nutritional deficiencies? Yes or No

Calude‐2: Yes, laparoscopic sleeve gastrectomy can lead to potential nutritional deficiencies in the long term. Because a portion of the stomach is removed during the procedure, the body′s ability to absorb some vitamins and minerals like iron, vitamin B12, folate, thiamine, and calcium can be reduced over time. Patients must closely follow their bariatric team′s nutritional guidelines after surgery, which include taking prescribed vitamin and mineral supplements daily, following up with blood work, and meeting protein intake recommendations. Most sleeve gastrectomy patients can avoid significant vitamin or mineral deficiencies after surgery with proper supplementation and nutritional counseling. However, non‐compliance with follow‐up care can increase the risks over the long run**.**

(Accessed dated December 21, 2023)

Chatbot responses are generally easier to read and understand than online materials [48], and chatbots can always be asked to explain matters in a simpler and easy‐to‐understand language. It is also worth mentioning that in the study by Ye et al., patients rated chatbot responses as easy to read as physician‐written responses to questions [39].

### 4.5. Reproducibility and Reliability

The reproducibility and reliability of answers varied significantly among different chatbots. The top‐performing chatbots, such as ChatGPT‐3.5 and ChatGPT‐4, showed superior reliability, achieving predominantly similar performance scores. Generally, chatbots demonstrated varying degrees of consistency, which underscores the importance of evaluating their performance in clinical contexts. This finding aligns with a previous study by Samaan et al., which reported similar reproducibility for ChatGPT; however, their analysis did not include other chatbots that demonstrated poor reliability and reproducibility in our study [38]. It is important to note that, currently, most chatbots lack the capability to retain the memory of previous queries, limiting their ability to offer personalized responses. However, recent developments indicate that this memory feature is being integrated into chatbots to enhance user experience and provide more tailored information. Future research should also evaluate the effect of retaining memory on the accuracy and helpfulness of chatbots for patients.

### 4.6. Future of Chatbots in Patient Education

Artificial intelligence’s growing prominence, especially LLMs and multimodal large language models (MLLM) in healthcare, has fueled discussions surrounding surpassing physicians in diverse facets of patient care. AI tools capable of managing patients’ entire journey, from providing information to making treatment decisions and facilitating personalized medicine and follow‐ups, are becoming increasingly relevant. However, the feasibility of AI overtaking physicians hinges on several factors, including the current capabilities of AI technology and the multifaceted nature of medical tasks.

Reflecting on past experiences, notably the “artificial intelligence winter” periods, we recognize the danger of unrealistic expectations surrounding AI’s capabilities. While the aspiration for AI tools with 100% accuracy in diagnosis and treatment, coupled with high levels of empathy, is commendable, achieving such a feat in the near future is unrealistic. Even if such a tool were developed, transitioning to and integrating it into healthcare systems would require time and consideration.

In this context, focusing on using AI as a complementary tool for patient education is a more achievable and practical goal. AI offers advantages such as round‐the‐clock availability and acceptable knowledge, which can complement physicians’ efforts in informing and educating patients. However, to employ AI’s full potential in this capacity, it is essential to establish specialized and standardized tests to evaluate and benchmark AI tools. For example, Barletta et al. have proposed a method of clinical chatbot evaluation based on the “quality in use” of ISO/IEC 25010. These approaches will ensure that AI’s contributions to patient care are effective and reliable [49].

While chatbots show promise in helping with patient education by providing accessible and often comprehensive information on medical inquiries, there are notable areas for improvement, such as empathy [50]. Additionally, translating these gains into real clinical encounters is nontrivial. Clinical consultations are interactive and context‐rich: how a question is phrased, clarified, and iteratively discussed—within active patient–clinician engagement and shared decision‐making—shapes which information is prioritized and how it is communicated. Effective use of chatbots also presupposes adequate digital and health literacy, as well as skill in formulating prompts; both vary widely across patients and may introduce inequities or miscommunication. Because chatbot outputs are sensitive to input wording and may overlook clinically salient nuance without guidance, their role should be to complement, not replace, clinical expertise. Accordingly, we position chatbots as adjuncts that can standardize and extend access to patient education while clinicians provide oversight to verify accuracy, tailor recommendations, and ensure empathetic, patient‐centered care.

### 4.7. Future Directions

Larger, multisite prospective evaluations that benchmark evolving AI tools against established standards of care are needed. Studies should implement embedded, clinician‐supervised deployments; measure behavioral and clinical outcomes (safety signals, delays in escalation, patient over‐reliance); assess medicolegal risk and governance (disclaimers, red‐flag routing, clinician sign‐off); use independent, blinded raters with separation of rubric authorship and scoring and Delphi‐finalized instruments, plus patient‐reported usefulness and task‐based endpoints; standardize patient‐phrased prompts in a single language and evaluate readability/health‐literacy and subgroup equity; and track model/versioning and citation/retrieval features.

### 4.8. Strengths and Limitations

This study focused on evaluating a broad set of prominent, general‐purpose chatbots as complementary, gap‐filling tools for bariatric patient education. Using a rigorous, clinically grounded FAQ—developed from international MBS society materials, real patient questions, and expert review—together with a structured answer key derived from MBS guidelines, we were able to assess the accuracy, completeness, and guideline‐concordance of chatbot responses in a meaningful way.

By including surgeons, fellows, and GPs (trained specifically for patient consultation for MBS in this center), and comparing these three clinician groups in subgroup analyses, we benchmarked chatbot performance against realistic patterns of information provision in everyday practice. We also incorporated several methodological innovations—domain‐level analyses, readability assessment, and test–retest reliability evaluation—offering a multidimensional and robust evaluation of chatbot performance.

While we included prominent chatbots, our research does not cover all available AI platforms. Our rating scale was designed for this study based on a guideline‐derived answer key and has not been previously validated or used in other studies. We did not quantify inter‐rater agreement, which may limit cross‐study comparability and introduce measurement error. Limited participant diversity and the single‐center design may restrict generalizability to other regions, care settings, and specialties, where practice patterns and thresholds may differ. The use of MISs as both rubric authors and raters may have biased scoring toward structured chatbot responses; future studies should separate these roles and incorporate independent raters and patient‐centered evaluations.

This study also did not test the ability of chatbots to simplify, rephrase, or further explain their answers upon prompting. A readability analysis was not performed for expert responses because they were produced in Persian, whereas chatbot outputs were evaluated in English, introducing potential cross‐language assessment bias. In addition, the questions were phrased by experts; how patients articulate queries (and their health literacy) can materially affect chatbot outputs. We also did not assess the effectiveness of institutional guardrails (disclaimers, red‐flag routing, oversight) that could mitigate misinformation and clarify medicolegal responsibility.

## 5. Conclusion

In this study, chatbots achieved higher accuracy and completeness scores than participating experts on patient questions. Given the realities of clinical care, chatbots should be viewed as complementary to clinical expertise, primarily by extending access to consistent patient education. Continued efforts to improve readability, reliability, and empathy, and to integrate these tools safely into clinical workflows, are essential for responsible adoption.

## Author Contributions

Amirreza Izadi: conceptualization, methodology, formal analysis, writing–original draft, and writing–review and editing.

Hesam Mosavari: conceptualization, methodology, writing–original draft, and writing–review and editing.

Ali Hosseininasab: writing–original draft and writing–review and editing.

Ali Jaliliyan: methodology and writing–review and editing.

Arzhang Jafari: data curation and writing–review and editing.

Mohammadhosein Akhlaghpasand: data curation and writing–review and editing.

Aghil Rostami: methodology and writing–review and editing.

Maziar Moradi‐Lakeh: methodology and writing–review and editing, supervision.

Foolad Eghbali: conceptualization and supervision.

## Funding

This work received no funding.

## Disclosure

A preprint has previously been published (DOI: 10.2196/preprints.67101). This manuscript is the most updated version, and it is not currently under consideration for peer review or publication in any other journal.

## Conflicts of Interest

The authors declare no conflicts of interest.

## Supporting Information

Table A1. Questions and answers used in the study.

Table A2. Pairwise comparison *p* values between groups in the lifestyle modification domain (ANOVA and Tukey’s post hoc tests).

Figure A1. Response distribution of each group in the lifestyle modification domain.

Figure A2. The performance score of each group in the lifestyle modification domain.

Table A3. Pairwise comparison *p* values between groups in the recovery and postoperative care domain (ANOVA and Tukey’s post hoc tests).

Figure A3. Response distribution of each group in the postoperative care domain.

Figure A4. The performance score of each group in the postoperative care domain.

Table A4. Pairwise comparison *p* values between groups in the risks and complications domain (ANOVA and Tukey’s post hoc tests).

Figure A5. Response distribution of each group in the risks and complication domain.

Figure A6. The performance score of each group in the outcome and expectation domain. MISs.

Table A5. Pairwise comparison *p* values between groups in the outcome and expectation domain (ANOVA and Tukey’s post hoc tests).

Figure A7. Response distribution of each group in the outcome and expectation domain.

Figure A8. The performance score of each group in the outcome and expectation domain.

Table A6. Pairwise comparison *p* values between groups in the preoperative care domain (ANOVA and Tukey’s post hoc tests).

Figure A9. Response distribution of each group in the preoperative care domain.

Figure A10. Performance score of each group in the preoperative care domain.

## Supporting information


**Supporting Information** Additional supporting information can be found online in the Supporting Information section.

## Data Availability

Data supporting the results of this study are available upon reasonable request from the corresponding author of this article.
