# Supplementary material for: Patient Education in Bariatric Surgery: Can Artificial Intelligence–Based Chatbots Bridge the Knowledge Gap?
Source: J Obes. 2026 Feb 12;2026:2376530. doi: 10.1155/jobe/2376530 (PMC12902178; doi:10.1155/jobe/2376530)
Supplement: Supplementary file 1 — Supporting Information Additional supporting information can be found online in the Supporting Information section. [file JOBE-2026-2376530-s001.zip › Supplementary file.docx]

**Supplementary file**

*Table A1: Questions and answers used in the study.*

| No | Q/A | Text | Category |
| --- | --- | --- | --- |
| 1 | Q | How much weight can I expect to lose 1-2 years after laparoscopic sleeve gastrectomy? (A range of percentage) | Outcomes & expectations |
|  | A | Excess weight loss (EWL) ranges from 50% to 70% [1]. | |
| 2 | Q | How long does it take to lose most of the weight after laparoscopic sleeve gastrectomy? | Outcomes & expectations |
|  | A | 6 months to 1 year [1]. | |
| 3 | Q | What are the criteria to qualify for laparoscopic sleeve gastrectomy? Maximum in two sentences | Pre-operative care |
|  | A | To qualify, individuals should have a BMI ≥ 40 kg/m2 or a BMI between 35 and 40 kg/m2 if they also have high-risk & complications comorbidities such as severe type 2 diabetes or cardiovascular Risks & complications factors 30 < BMI < 35 with diabetes that can’t be controlled by medication [1]. | |
| 4 | Q | What are the four conditions or comorbidities that may improve after laparoscopic sleeve gastrectomy? | Outcomes & expectations |
|  | A | LSG improves or rehabilitates type 2 diabetes, hypertension, dyslipidemia, sleep apnea, and other weight-related diseases [1]. | |
| 5 | Q | Mention four medications that should stop before laparoscopic sleeve gastrectomy. | Pre-operative care |
|  | A | Patients are advised to discuss medication management with their surgical team, who will provide instructions on any required preoperative medication adjustments. Four medications: NSAIDs, OCP, Warfarin, Plavix, and some antihyperglycemics should be stopped before the operation [1]. | |
| 6 | Q | What type of dietary changes are required before laparoscopic sleeve gastrectomy? Maximum in two sentences | Pre-operative care |
|  | A | Before bariatric surgery, preoperative weight loss using a low or low-calorie diet should be recommended. Solids until six hours before induction and clear liquids until two hours before induction for elective bariatric surgery assuming no contraindications [2]. | |
| 7 | Q | What are the steps involved in preparing for laparoscopic sleeve gastrectomy? Maximum in 3 sentences | Pre-operative care |
|  | A | Complete History & Physical examination, Routine labs, Nutrient screening, Cardiopulmonary evaluation, GI evaluation, Endocrine evaluation, Lifestyle medicine evaluation, Clinical nutrition evaluation, Psychosocial-behavioral evaluation [3]. | |
| 8 | Q | What do I need to do the day before laparoscopic sleeve gastrectomy to get ready? Maximum in 3 sentences | Pre-operative care |
|  | A | Patients are recommended to fast after midnight, arrive at the recommended time, bring a medication list, and follow surgeon instructions for the day before surgery [1]. | |
| 9 | Q | How long is the hospital stay after laparoscopic sleeve gastrectomy? A range of days | Recovery & post-operative care |
|  | A | Hospital stay is typically 1-3 days [1]. | |
| 10 | Q | What are the major Risks & complicationss and complications of laparoscopic sleeve gastrectomy? Maximum in 3 sentences | Risks & complications |
|  | A | Leaks, bleeding, blood clots, infection, nausea, GERD symptoms, stricture, and nutritional deficiencies are potential Risks & complicationss and complications after sleeve gastrectomy [1]. | |
| 11 | Q | How common are serious complications like leaks at the staple line after laparoscopic sleeve gastrectomy? A range of percentage | Risks & complications |
|  | A | Staple line leaks occur in about 0.9-2.2% of sleeve gastrectomy patients [4]. | |
| 12 | Q | What is the mortality Risks & complications with laparoscopic sleeve gastrectomy? A range of percentage | Risks & complications |
|  | A | The mortality Risks & complications is very low at 0-3.3%% with sleeve gastrectomy [4]. | |
| 13 | Q | Can laparoscopic sleeve gastrectomy lead to long-term nutritional deficiencies? Yes or no | Risks & complications |
|  | A | Without supplementation, vitamin and mineral deficiencies can occur long-term after sleeve gastrectomy [3]. | |
| 14 | Q | Can laparoscopic sleeve gastrectomy be revised or converted to another bariatric surgery if inadequate weight loss occurs? Yes or no | Outcomes & expectations |
|  | A | Yes, revisional bariatric surgery is an option if inadequate weight loss or weight regain occurs after the initial sleeve procedure [4]. | |
| 15 | Q | When can I resume normal daily activities and lift permission after laparoscopic sleeve gastrectomy? Two separate ranges of weeks | Recovery & post-operative care |
|  | A | Light daily activities can resume within 1-2 weeks, with lifting restricted for 4-6 weeks postoperatively [5]. | |
| 16 | Q | What severity of pain or discomfort is typical after laparoscopic sleeve gastrectomy? Maximum two words | Recovery & post-operative care |
|  | A | Shoulder pain, abdominal soreness, fullness, and cramping are joint but usually mild by 4-5 days after LSG [2]. | |
| 17 | Q | What medications will be prescribed after discharge from the hospital for laparoscopic sleeve gastrectomy? Maximum 10 Drug Categories | Recovery & post-operative care |
|  | A | Analgesics, anti-nausea medication, acid-reducing drugs, and prophylactic blood thinners are recommended after surgery as needed [1]. | |
| 18 | Q | How long until I can start exercising after laparoscopic sleeve gastrectomy? Range of weeks | Recovery & post-operative care |
|  | A | 1 week start, 6–8-week hard physical activity [Internal protocol^a^]. There is still no definite time to start exercise after bariatric surgery in existing evidence. Protocols differ from 7 days after surgery to 6 months after that and even later. However, patients can begin physical activity from the day of surgery by walking short distances and going out of bed. A structured, individualized exercise program could start a week after surgery when the patient is discharged and does not need medical supervision [5]. | |
| 19 | Q | When are follow-up appointments required after laparoscopic sleeve gastrectomy with my bariatric surgeon? Maximum ten intervals | Recovery & post-operative care |
|  | A | After LSG, follow-ups are recommended every week, one month, three months, six months, twelve months, and then annually [3]. | |
| 20 | Q | How long until I can return to work after laparoscopic sleeve gastrectomy? Range of months | Recovery & post-operative care |
|  | A | After surgery, most patients return to work in one or two weeks [6, 7]. | |
| 21 | Q | How does eating change after laparoscopic sleeve gastrectomy? Maximum three sentences | Life-style modifications |
|  | A | A well-balanced diet containing all of the essential nutrients is recommended for continued good health and weight maintenance. Patients should consume fluids slowly and avoid drinking 30 minutes before or after meals to prevent gastrointestinal symptoms. Adequate hydration is critical, with a recommended intake of more than 1.5 liters daily. Practice mindful eating. Chew all food until it is smooth. Make sure food is soft and moist enough to swallow without sticking. Avoid bread, rice, and pasta until able to comfortably consume adequate protein, vegetables, and fruits [3]. | |
| 22 | Q | What is a sample menu for the first month after laparoscopic sleeve gastrectomy? Maximum three sentences | Recovery & post-operative care |
|  | A | Solid foods with an emphasis on protein sources, some carbohydrates, and fiber (~10-14 days after surgery) Micronutrient supplementation (when the patient reaches a stable or maintenance Weight). Keep sipping fluid 48-64 ounces daily (continue sugar-free, non-carbonated choices). Use this meal plan as a guide for a balanced diet, focusing on protein first (at least 60-70 g daily). Keep taking vitamin and mineral supplements; you can change to pill form at this stage if you prefer [3]. | |
| 23 | Q | Are there any food restrictions right after laparoscopic sleeve gastrectomy? Yes or no | Recovery & post-operative care |
|  | A | Patients should avoid solid foods while the stomach is healing by carefully following the surgeon's prescribed diet instructions. The diet progresses from clear to full liquids to pureed foods over four weeks postoperatively [6, 7]. | |
| 24 | Q | How many meals per day will I be eating after laparoscopic sleeve gastrectomy? Range of numbers | Life-style modifications |
|  | A | Patients should be counseled to eat three small meals daily and chew small bites thoroughly before swallowing. Patients should be counseled about the principles of healthy eating, including at least five daily servings of fresh fruits and vegetables [3]. | |
| 25 | Q | Can I drink carbonated beverages after laparoscopic sleeve gastrectomy? Maximum one sentences | Life-style modifications |
|  | A | Carbonation can cause discomfort, so it should be limited if tolerated. ASMBS suggests avoiding and delay using of carbonated beverages [3]. | |
| 26 | Q | Are protein supplements recommended after laparoscopic sleeve gastrectomy? Yes or no | Life-style modifications |
|  | A | ASMBS recommends daily supplements, 60-100 g of protein [8]. | |
| 27 | Q | What are strategies to ensure I get enough nutrients after laparoscopic sleeve gastrectomy? Maximum three sentences | Life-style modifications |
|  | A | ASMBS recommends supplements, 60-100g of protein daily, increased fluids, a balanced diet with fruits and vegetables, and follow-up lab testing to ensure adequate nutrition, Laboratory markers are considered imperative for completing the initial nutrition assessment and follow-up for surgical weight loss patients. Established baseline values are important when trying to distinguish between postoperative complications, deficiencies related to surgery, noncompliance with recommended nutrient supplementation, or nutritional complications arising from preexisting deficiencies. Additional laboratory measures might be required and are defined by the presence of the existing individual co-morbid conditions [8]. | |
| 28 | Q | How long until I can resume alcohol consumption after laparoscopic sleeve gastrectomy? Range of weeks, months, or years | Life-style modifications |
|  | A | Patients are advised to avoid alcohol for at least 6-12 months or until reaching the goal weight after LSG [4] [Internal protocol^a^]. | |
| 29 | Q | What lifestyle changes are critical for success after laparoscopic sleeve gastrectomy? Maximum three sentences | Life-style modifications |
|  | A | Dietary adherence, regular exercise, taking supplements, keeping appointments, behavior modification, and joining support groups lead to better bariatric surgery outcomes [1]. | |
| 30 | Q | Does laparoscopic sleeve gastrectomy reverse type 2 diabetes? If so, how much and how quickly? Maximum three sentences | Outcomes & expectations |
|  | A | Yes, LSG results in the remission of type 2 diabetes in 50-90% of patients, often within days to weeks postoperatively [1]. | |
| 31 | Q | Does the laparoscopic sleeve gastrectomy improve fertility or outcomes in pregnancy? Yes or no | Outcomes & expectations |
|  | A | Yes, LSG may improve fertility and pregnancy outcomes [1]. | |
| 32 | Q | What kinds of exercise provide the most benefit after laparoscopic sleeve gastrectomy? Maximum three sentences | Life-style modifications |
|  | A | After weight loss surgery, patients often feel they have more energy and may want to exercise. Walking frequently, starting just a few hours after surgery, can help patients recover faster. Each patient will be different and should check with their surgeon before they begin intense exercise, but once this is started, it should be done with the goal of an average of 30 minutes per day of moderate exercise [9]. | |
| 33 | Q | Will I need plastic surgery for excess skin after reaching my goal weight after laparoscopic sleeve gastrectomy? Maximum two sentences | Outcomes & expectations |
|  | A | That is not always the case. As a rule, plastic surgery will not be considered for at least a year or two after the operation. Sometimes, the skin will mold itself around the new body tissue. It would be best to give the skin the time it needs to adjust before you decide to have more surgery [7]. | |
| 34 | Q | How long are vitamin supplements required after laparoscopic sleeve gastrectomy? Yes or no | Life-style modifications |
|  | A | You may. It’s possible you may not get enough vitamins from three small meals a day. At your regular check-ups, your specialist will evaluate whether you are getting enough vitamin B12, folic acid, and iron [7]. | |
| 35 | Q | What type of scarring results from laparoscopic sleeve gastrectomy surgery? | Risks & complications |
|  | A | Five incisions 5 mm-15 mm above the umbilicus [1]. | |
| 36 | Q | Is laparoscopic sleeve gastrectomy reversible if desired? Yes or no | Outcomes & expectations |
|  | A | No, LSG is not easily reversible since it involves surgical removal of stomach tissue [4]. | |
| 37 | Q | How does laparoscopic sleeve gastrectomy work to achieve weight loss? Maximum three sentences | Outcomes & expectations |
|  | A | LSG restricts food intake and suppresses hunger hormones like ghrelin to promote weight loss [1]. | |
| 38 | Q | Will health insurance cover the costs of laparoscopic sleeve gastrectomy if BMI is 42 kg/m2? Yes or no | Pre-operative care |
|  | A | Many insurance companies cover LSG with preauthorization, especially for patients who meet NIH criteria [10]. | |
| 39 | Q | Is there an increased Risks & complications of substance abuse after laparoscopic sleeve gastrectomy? Yes or no | Risks & complications |
|  | A | Postoperative bariatric surgery patients are at a higher Risks & complications of alcohol use disorders (AUD) compared to the general population. The Longitudinal Assessment of Bariatric Surgery-2 (LABS-2) demonstrated a twofold increased Risks & complications for AUD and an increased Risks & complications for SUD after bariatric surgery [4]. | |
| 40 | Q | What support resources are available after laparoscopic sleeve gastrectomy? Maximum two sentences | Risks & complications |
|  | A | Social workers: A licensed social worker is uniquely qualified to address these broad and diverse factors. While not a required team member, the social worker can contribute to optimizing the quality-of-care patients receive throughout the bariatric surgery process. Familial Support, Social Support, Economic Support [4]. | |

*^a^ Internal protocol refers to the protocol used in Rasoul-e-Akram Hospital, an International Federation for the Surgery of Obesity and Metabolic Disorders Center of Excellence (IFSO-COE) for metabolic and bariatric surgery.*

**References for Appendix 1**

1. Zundel N, Hernandez R. JD, Gagner M. Laparoscopic Sleeve Gastrectomy: Technique and Outcomes. In: Nguyen NT, Brethauer SA, Morton JM, Ponce J, Rosenthal RJ, editors. ASMBS Textb Bariatr Surg Cham: Springer International Publishing; 2020. p. 149–159. doi: 10.1007/978-3-030-27021-6_13ISBN:978-3-030-27020-9

2. Stenberg E, Dos Reis Falcão LF, O’Kane M, Liem R, Pournaras DJ, Salminen P, Urman RD, Wadhwa A, Gustafsson UO, Thorell A. Guidelines for Perioperative Care in Bariatric Surgery: Enhanced Recovery After Surgery (ERAS) Society Recommendations: A 2021 Update. World J Surg 2022 Apr;46(4):729–751. doi: 10.1007/s00268-021-06394-9

3. Mechanick JI, Apovian C, Brethauer S, Timothy Garvey W, Joffe AM, Kim J, Kushner RF, Lindquist R, Pessah‐Pollack R, Seger J, Urman RD, Adams S, Cleek JB, Correa R, Figaro MK, Flanders K, Grams J, Hurley DL, Kothari S, Seger MV, Still CD. Clinical Practice Guidelines for the Perioperative Nutrition, Metabolic, and Nonsurgical Support of Patients Undergoing Bariatric Procedures – 2019 Update: Cosponsored by American Association of Clinical Endocrinologists/American College of Endocrinology, The Obesity Society, American Society for Metabolic and Bariatric Surgery, Obesity Medicine Association, and American Society of Anesthesiologists. Obesity 2020 Apr;28(4). doi: 10.1002/oby.22719

4. Reavis KM, Barrett AM, Kroh MD, editors. The SAGES Manual of Bariatric Surgery. second. Cham, Switzerland: Springer Cham; 2018. ISBN:978-3-319-71281-9

5. Tabesh MR, Eghtesadi M, Abolhasani M, Maleklou F, Ejtehadi F, Alizadeh Z. Nutrition, Physical Activity, and Prescription of Supplements in Pre- and Post-bariatric Surgery Patients: An Updated Comprehensive Practical Guideline. Obes Surg 2023 Aug;33(8):2557–2572. doi: 10.1007/s11695-023-06703-2

6. ASMBS Public Education Committee. FAQs of Bariatric Surgery. FAQs Bariatr Surg. 2020. Available from: https://asmbs.org/patients/faqs-of-bariatric-surgery/ [accessed Nov 2, 2023]

7. IFSO. FAQ on Obesity Surgery. FAQ Obes Surg. 2023. Available from: https://www.ifso.com/faq-obesity-surgery/ [accessed Nov 2, 2023]

8. Aills L, Blankenship J, Buffington C, Furtado M, Parrott J. ASMBS Allied Health Nutritional Guidelines for the Surgical Weight Loss Patient. Surg Obes Relat Dis 2008 Sep;4(5):S73–S108. doi: 10.1016/j.soard.2008.03.002

9. ASMBS Public Education Committee. Life After Bariatric Surgery. Life Bariatr Surg. 2021. Available from: https://asmbs.org/patients/life-after-bariatric-surgery/ [accessed Nov 2, 2023]

10. Eisenberg D, Shikora SA, Aarts E, Aminian A, Angrisani L, Cohen RV, De Luca M, Faria SL, Goodpaster KPS, Haddad A, Himpens JM, Kow L, Kurian M, Loi K, Mahawar K, Nimeri A, O’Kane M, Papasavas PK, Ponce J, Pratt JSA, Rogers AM, Steele KE, Suter M, Kothari SN. 2022 American Society of Metabolic and Bariatric Surgery (ASMBS) and International Federation for the Surgery of Obesity and Metabolic Disorders (IFSO) Indications for Metabolic and Bariatric Surgery. Obes Surg 2023 Jan;33(1):3–14. doi: 10.1007/s11695-022-06332-1

*Table A2. Pairwise comparison p-values between groups in the lifestyle modification domain (ANOVA and Tukey's post hoc tests).*

|  | MISs^a^ | MIFs | GPs | Bing | Bard | Claude | Llama | Perplexity | ChatGPT-3.5 |
| --- | --- | --- | --- | --- | --- | --- | --- | --- | --- |
| MIFs^b^ | 0.483 |  |  |  |  |  |  |  |  |
| GPs^c^ | 0.585 | 1.000 |  |  |  |  |  |  |  |
| Bing | 1.000 | 0.989 | 0.957 |  |  |  |  |  |  |
| Bard | 0.987 | 0.193 | 0.259 | 0.910 |  |  |  |  |  |
| Claude | 1.000 | 0.661 | 0.645 | 0.999 | 0.999 |  |  |  |  |
| Llama | 0.905 | 1.000 | 1.000 | 0.999 | 0.537 | 0.910 |  |  |  |
| Perplexity | 0.987 | 1.000 | 0.995 | 1.000 | 0.748 | 0.984 | 1.000 |  |  |
| ChatGPT-3.5 | 0.960 | 0.129 | 0.194 | 0.839 | 1.000 | 0.995 | 0.433 | 0.645 |  |
| ChatGPT-4 | 0.960 | 0.129 | 0.194 | 0.839 | 1.000 | 0.995 | 0.433 | 0.645 | 1.000 |

*^a^Minimally Invasive Surgeons, ^b^Minimally Invasive Surgery Fellows, ^c^General Practitioners,*

** Statistically significant*

*Figure A1. Responses distribution of each group in the lifestyle modification domain. MISs, Minimally invasive surgeons; MIFs, Minimally invasive surgery fellows; GPs, General practitioners.*

**

*Figure A2. The performance score of each group in the lifestyle modification domain.* *MISs, Minimally invasive surgeons; MIFs, Minimally invasive surgery fellows; GPs, General practitioners.*

*Table A3. Pairwise comparison p-values between groups in the recovery and post-operative care domain (ANOVA and Tukey's post hoc tests).*

|  | MISs^a^ | MIFs | GPs | Bing | Bard | Claude | Llama | Perplexity | ChatGPT-3.5 |
| --- | --- | --- | --- | --- | --- | --- | --- | --- | --- |
| MIFs^b^ | 0.213 |  |  |  |  |  |  |  |  |
| GPs^c^ | 0.845 | 1.000 |  |  |  |  |  |  |  |
| Bing | 0.845 | 1.000 | 1.000 |  |  |  |  |  |  |
| Bard | 1.000 | 0.910 | 0.997 | 0.997 |  |  |  |  |  |
| Claude | 1.000 | 0.910 | 0.997 | 0.997 | 1.000 |  |  |  |  |
| Llama | 0.998 | 0.957 | 0.999 | 0.999 | 1.000 | 1.000 |  |  |  |
| Perplexity | 1.000 | 0.750 | 0.980 | 0.980 | 1.000 | 1.000 | 1.000 |  |  |
| ChatGPT-3.5 | 1.000 | 0.341 | 0.815 | 0.815 | 0.997 | 0.997 | 0.992 | 1.000 |  |
| ChatGPT-4 | 1.000 | 0.261 | 0.743 | 0.743 | 0.992 | 0.992 | 0.980 | 0.999 | 1.000 |

*^a^Minimally Invasive Surgeons, ^b^Minimally Invasive Surgery Fellows, ^c^General Practitioners*

** Statistically significant*

*Figure A3. Responses distribution of each group in the post-operative care domain. MISs, Minimally invasive surgeons; MIFs, Minimally invasive surgery fellows; GPs, General practitioners.*

*Figure A4. The performance score of each group in the post-operative care domain.* *MISs, Minimally invasive surgeons; MIFs, Minimally invasive surgery fellows; GPs, General practitioners.*

*Table A4. Pairwise comparison p-values between groups in the risks and complications domain (ANOVA and Tukey's post hoc tests).*

|  | MISs^a^ | MIFs | GPs | Bing | Bard | Claude | Llama | Perplexity | ChatGPT-3.5 |
| --- | --- | --- | --- | --- | --- | --- | --- | --- | --- |
| MIFs^b^ | 0.307 |  |  |  |  |  |  |  |  |
| GPs^c^ | 1.000 | 0.914 |  |  |  |  |  |  |  |
| Bing | 0.998 | 0.190 | 0.978 |  |  |  |  |  |  |
| Bard | 0.976 | 0.999 | 1.000 | 0.800 |  |  |  |  |  |
| Claude | 1.000 | 0.337 | 0.997 | 1.000 | 0.917 |  |  |  |  |
| Llama | 0.991 | 0.996 | 1.000 | 0.865 | 1.000 | 0.954 |  |  |  |
| Perplexity | 1.000 | 0.647 | 1.000 | 0.999 | 0.991 | 1.000 | 0.997 |  |  |
| ChatGPT-3.5 | 0.898 | 0.049* | 0.800 | 1.000 | 0.473 | 0.997 | 0.557 | 0.954 |  |
| ChatGPT-4 | 0.898 | 0.049* | 0.800 | 1.000 | 0.473 | 0.997 | 0.557 | 0.954 | 1.000 |

*^a^Minimally Invasive Surgeons, ^b^Minimally Invasive Surgery Fellows, ^c^General Practitioners*

** Statistically significant*

*Figure A5. Responses distribution of each group in the risks and complications domain. MISs, Minimally invasive surgeons; MIFs, Minimally invasive surgery fellows; GPs, General practitioners.*

*Figure A6. The performance score of each group in the outcome and expectation domain. MISs, Minimally invasive surgeons; MIFs, Minimally invasive surgery fellows; GPs, General practitioners.*

*Table A5. Pairwise comparison p-values between groups in the outcome and expectation domain (ANOVA and Tukey's post hoc tests).*

|  | MISs^a^ | MIFs | GPs | Bing | Bard | Claude | Llama | Perplexity | ChatGPT-3.5 |
| --- | --- | --- | --- | --- | --- | --- | --- | --- | --- |
| MIFs^b^ | 0.999 |  |  |  |  |  |  |  |  |
| GPs^c^ | 1.000 | 1.000 |  |  |  |  |  |  |  |
| Bing | 0.986 | 0.768 | 0.985 |  |  |  |  |  |  |
| Bard | 0.774 | 0.327 | 0.816 | 1.000 |  |  |  |  |  |
| Claude | 0.676 | 0.243 | 0.737 | 0.999 | 1.000 |  |  |  |  |
| Llama | 0.986 | 0.768 | 0.985 | 1.000 | 1.000 | 0.999 |  |  |  |
| Perplexity | 0.999 | 0.930 | 0.999 | 1.000 | 0.995 | 0.985 | 1.000 |  |  |
| ChatGPT-3.5 | 0.22 | 0.04* | 0.311 | 0.882 | 0.995 | 0.999 | 0.882 | 0.737 |  |
| ChatGPT-4 | 0.29 | 0.059 | 0.386 | 0.932 | 0.999 | 1.000 | 0.932 | 0.816 | 1.000 |

*^a^Minimally Invasive Surgeons, ^b^Minimally Invasive Surgery Fellows, ^c^General Practitioners*

** Statistically significant*

*Figure A7. Responses distribution of each group in the outcome and expectation domain. MISs, Minimally invasive surgeons; MIFs, Minimally invasive surgery fellows; GPs, General practitioners.*

*Figure A8. The performance score of each group in the outcome and expectation domain. MISs, Minimally invasive surgeons; MIFs, Minimally invasive surgery fellows; GPs, General practitioners.*

*Table A6. Pairwise comparison p-values between groups in the pre-operative care domain (ANOVA and Tukey's post hoc tests).*

|  | MISs^a^ | MIFs | GPs | Bing | Bard | Claude | Llama | Perplexity | ChatGPT-3.5 |
| --- | --- | --- | --- | --- | --- | --- | --- | --- | --- |
| MIFs^b^ | 0.07 |  |  |  |  |  |  |  |  |
| GPs^c^ | 0.709 | 1.000 |  |  |  |  |  |  |  |
| Bing | 0.861 | 0.01 | 0.164 |  |  |  |  |  |  |
| Bard | 1.000 | 0.146 | 0.667 | 0.988 |  |  |  |  |  |
| Claude | 0.979 | 0.029 | 0.306 | 1.000 | 1.000 |  |  |  |  |
| Llama | 0.999 | 0.707 | 0.988 | 0.667 | 0.995 | .867 |  |  |  |
| Perplexity | 1.000 | 0.108 | 0.589 | 0.995 | 1.000 | 1.000 | 0.988 |  |  |
| ChatGPT-3.5 | 0.791 | 0.007* | 0.130 | 1.000 | 0.973 | 1.000 | 0.589 | 0.988 |  |
| ChatGPT-4 | 0.621 | 0.003* | 0.081 | 1.000 | 0.915 | 0.998 | 0.438 | 0.950 | 1.000 |

*^a^Minimally Invasive Surgeons, ^b^Minimally Invasive Surgery Fellows, ^c^General Practitioners*

** Statistically significant*

*Figure A9. Response distribution of each group in the pre-operative care domain. MISs, Minimally invasive surgeons; MIFs, Minimally invasive surgery fellows; GPs, General practitioners.*

*Figure A10. Performance score of each group in the pre-operative care domain. MISs, Minimally invasive surgeons; MIFs, Minimally invasive surgery fellows; GPs, General practitioners.*
