# Supplementary figures and images for: Patient Education in Bariatric Surgery: Can Artificial Intelligence–Based Chatbots Bridge the Knowledge Gap?
Source: J Obes. 2026 Feb 12;2026:2376530. doi: 10.1155/jobe/2376530 (PMC12902178; doi:10.1155/jobe/2376530)

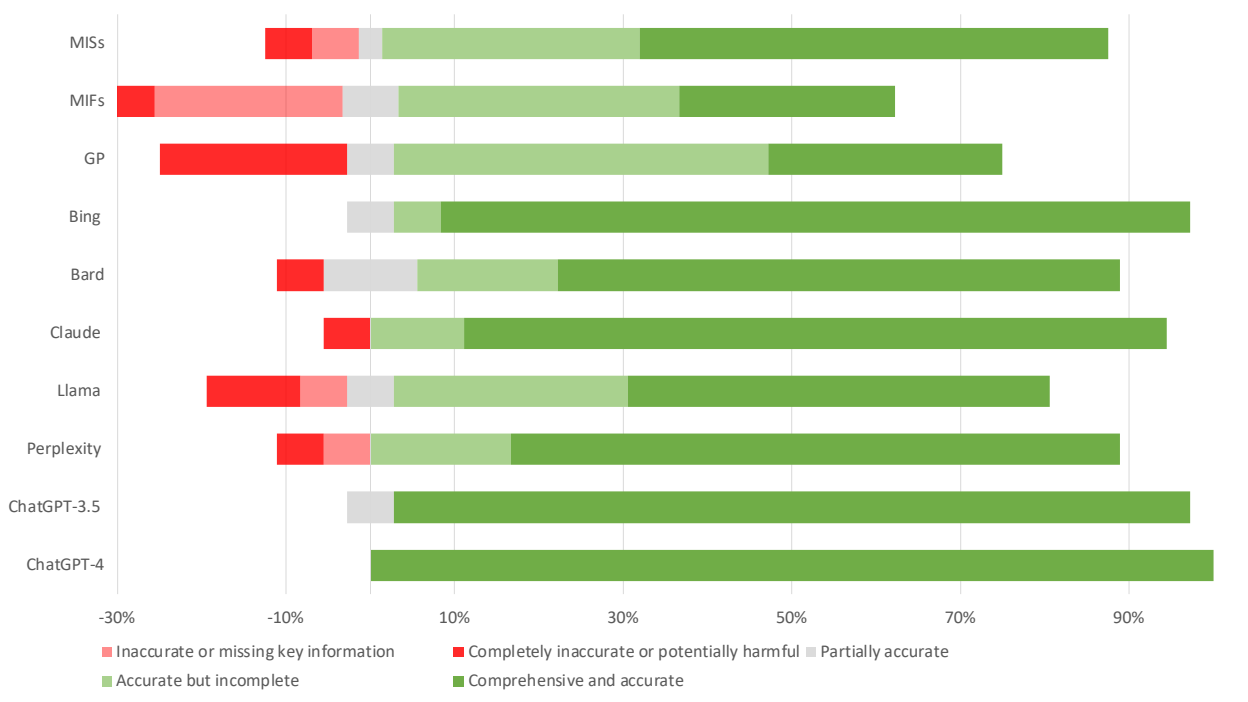

Supplement: Supplementary file 1 — Supporting Information Additional supporting information can be found online in the Supporting Information section. [file JOBE-2026-2376530-s001.zip › Fig-A1.pdf]

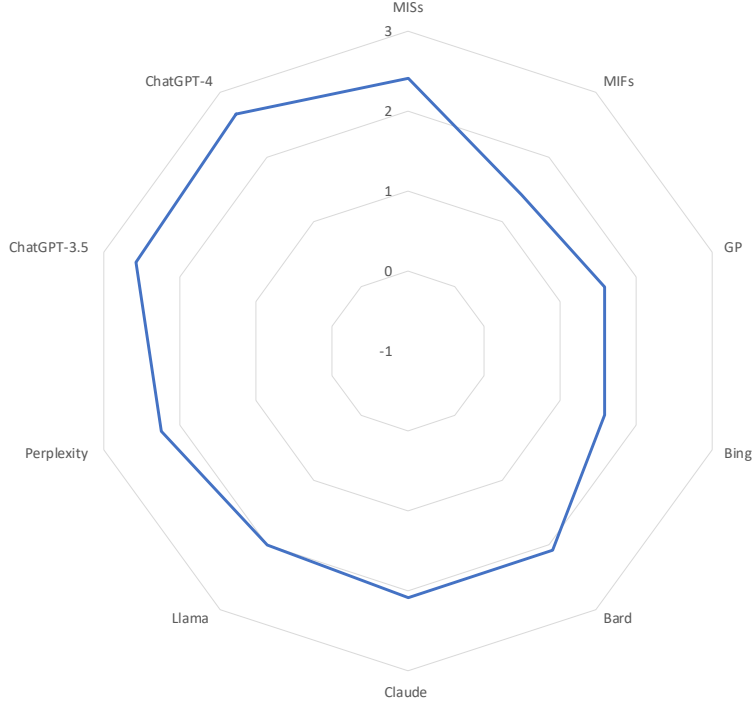

Supplement: Supplementary file 1 — Supporting Information Additional supporting information can be found online in the Supporting Information section. [file JOBE-2026-2376530-s001.zip › Fig-A10.pdf]

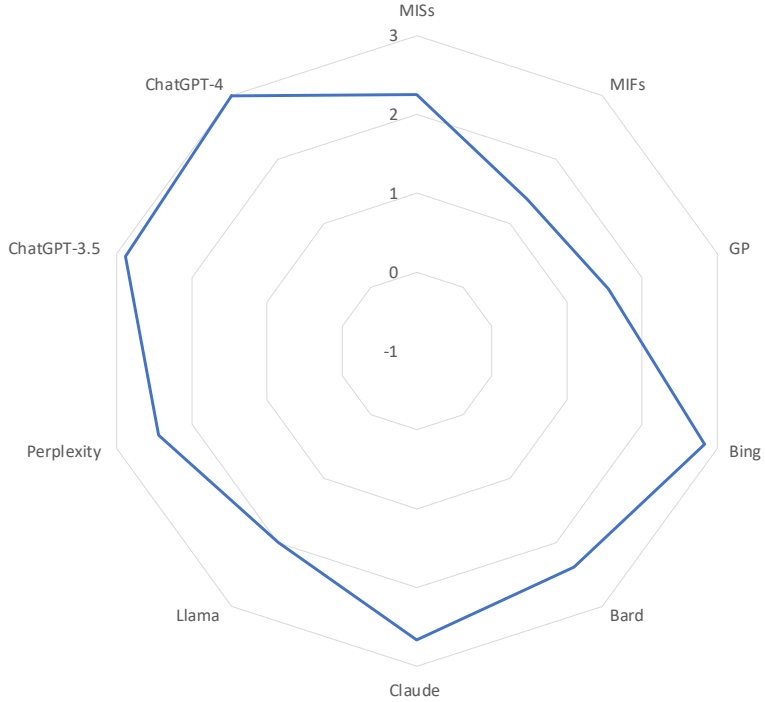

Supplement: Supplementary file 1 — Supporting Information Additional supporting information can be found online in the Supporting Information section. [file JOBE-2026-2376530-s001.zip › Fig-A2.pdf]

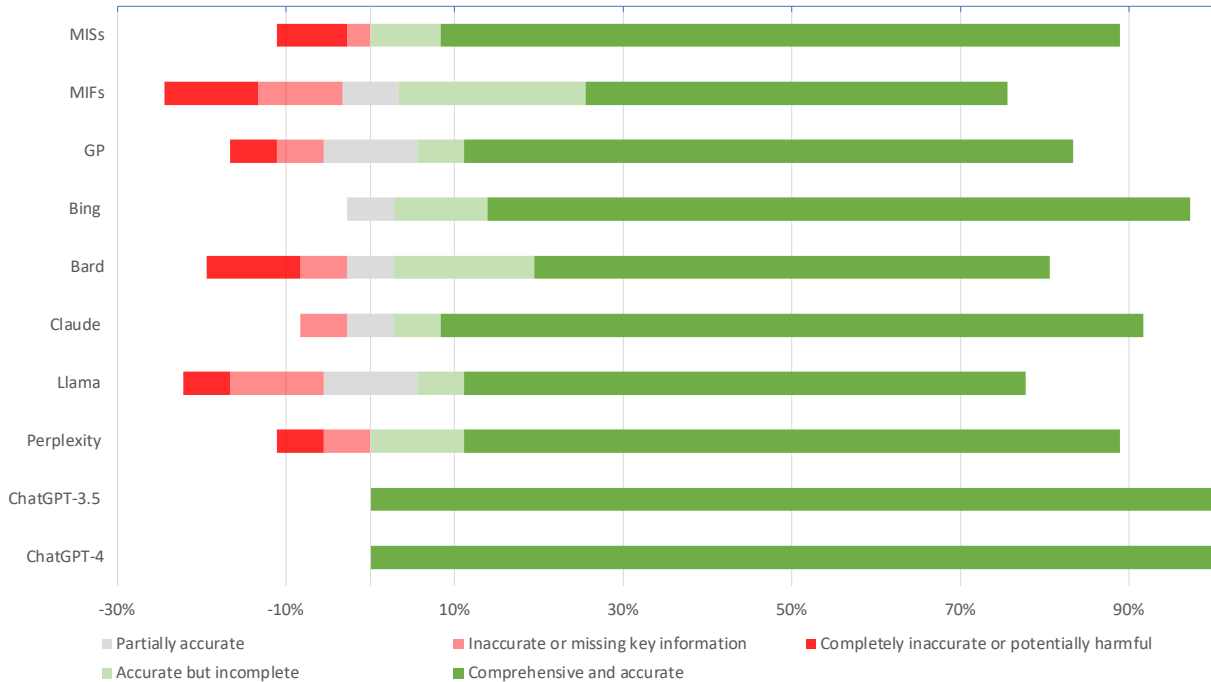

Supplement: Supplementary file 1 — Supporting Information Additional supporting information can be found online in the Supporting Information section. [file JOBE-2026-2376530-s001.zip › Fig-A3.pdf]

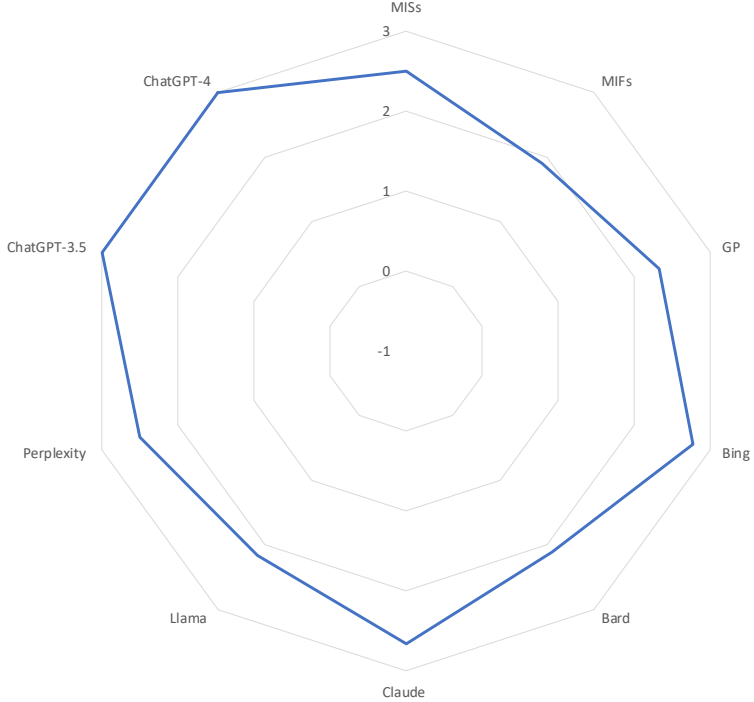

Supplement: Supplementary file 1 — Supporting Information Additional supporting information can be found online in the Supporting Information section. [file JOBE-2026-2376530-s001.zip › Fig-A4.pdf]

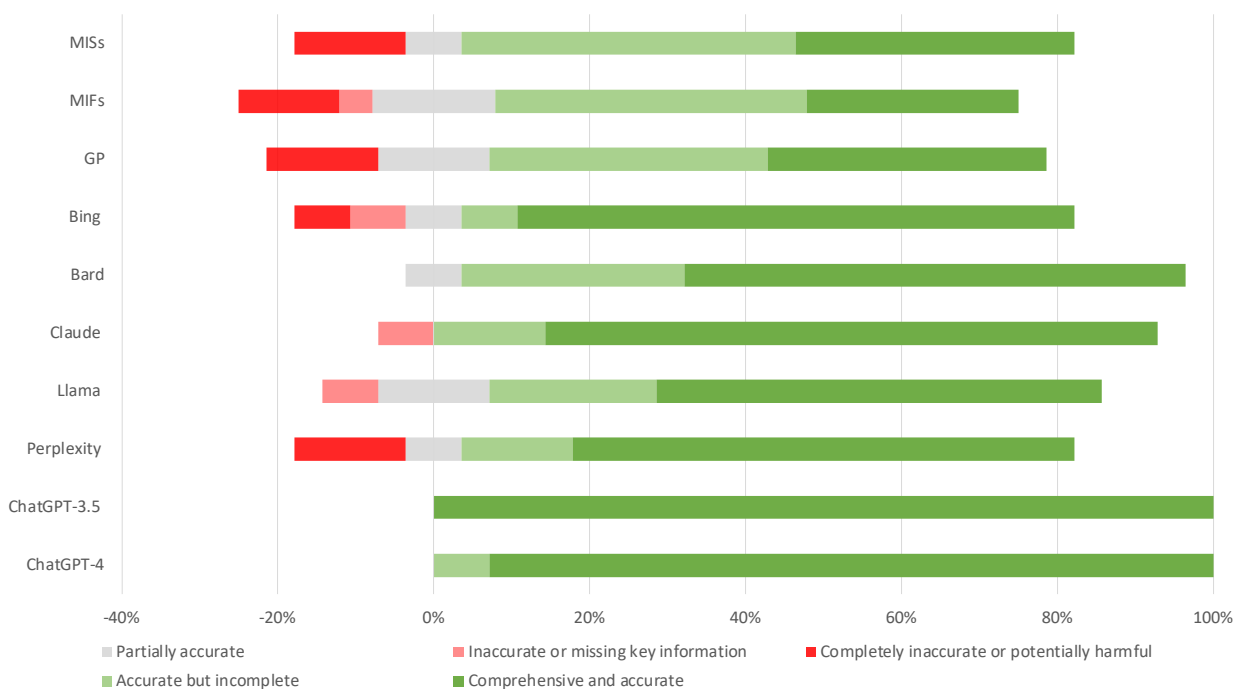

Supplement: Supplementary file 1 — Supporting Information Additional supporting information can be found online in the Supporting Information section. [file JOBE-2026-2376530-s001.zip › Fig-A5.pdf]

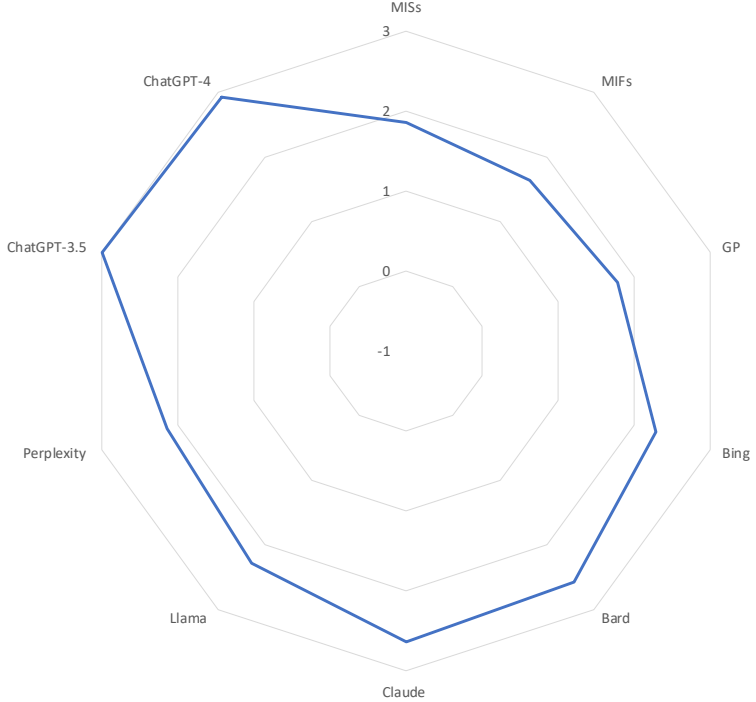

Supplement: Supplementary file 1 — Supporting Information Additional supporting information can be found online in the Supporting Information section. [file JOBE-2026-2376530-s001.zip › Fig-A6.pdf]

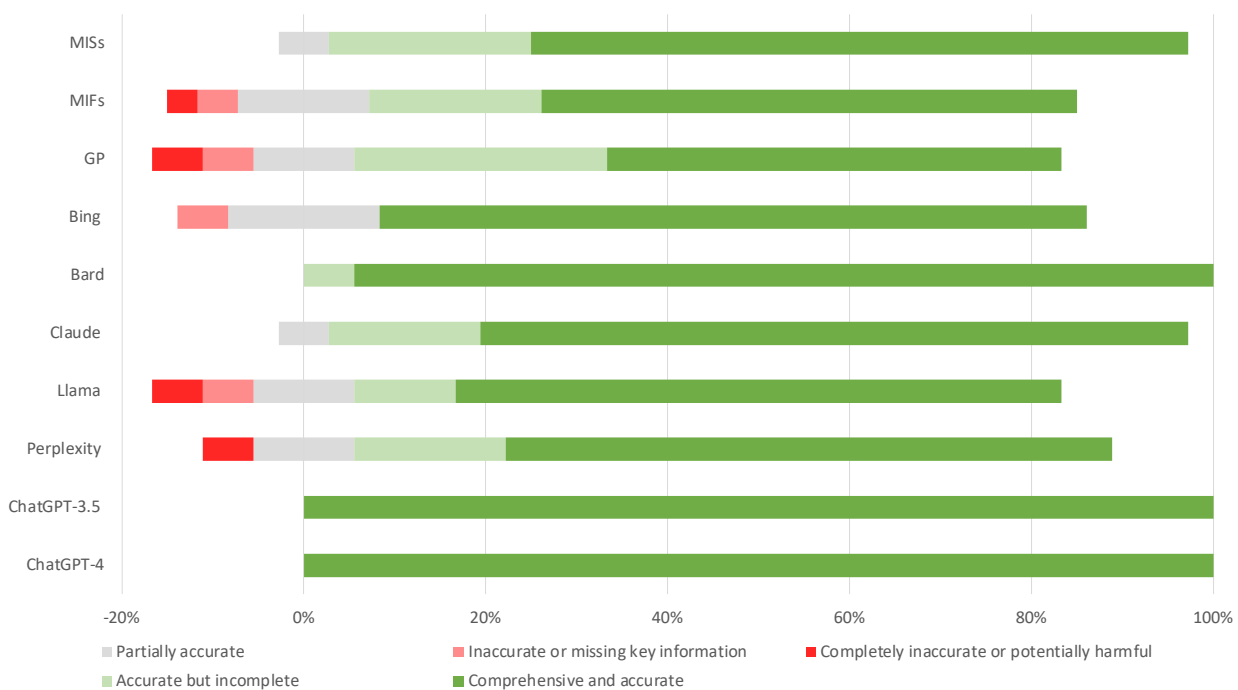

Supplement: Supplementary file 1 — Supporting Information Additional supporting information can be found online in the Supporting Information section. [file JOBE-2026-2376530-s001.zip › Fig-A7.pdf]

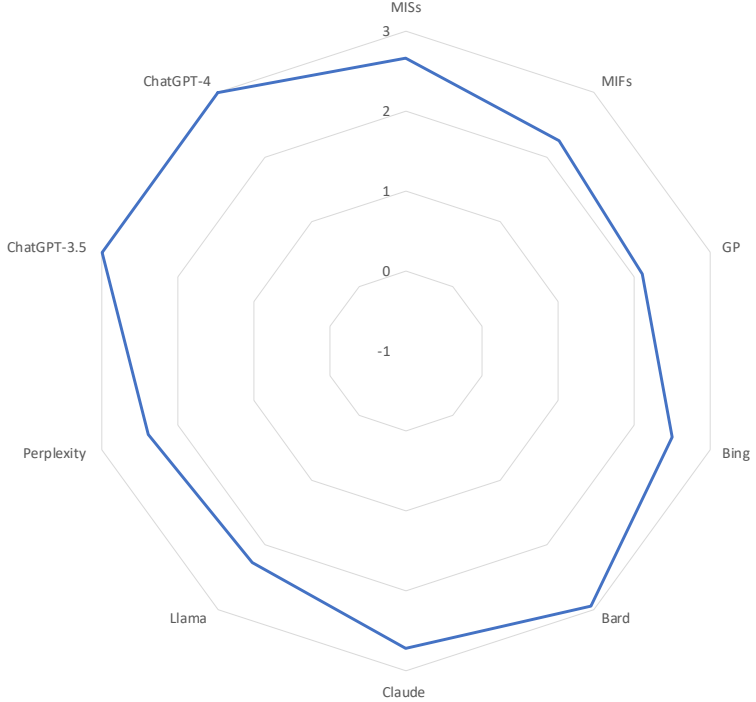

Supplement: Supplementary file 1 — Supporting Information Additional supporting information can be found online in the Supporting Information section. [file JOBE-2026-2376530-s001.zip › Fig-A8.pdf]

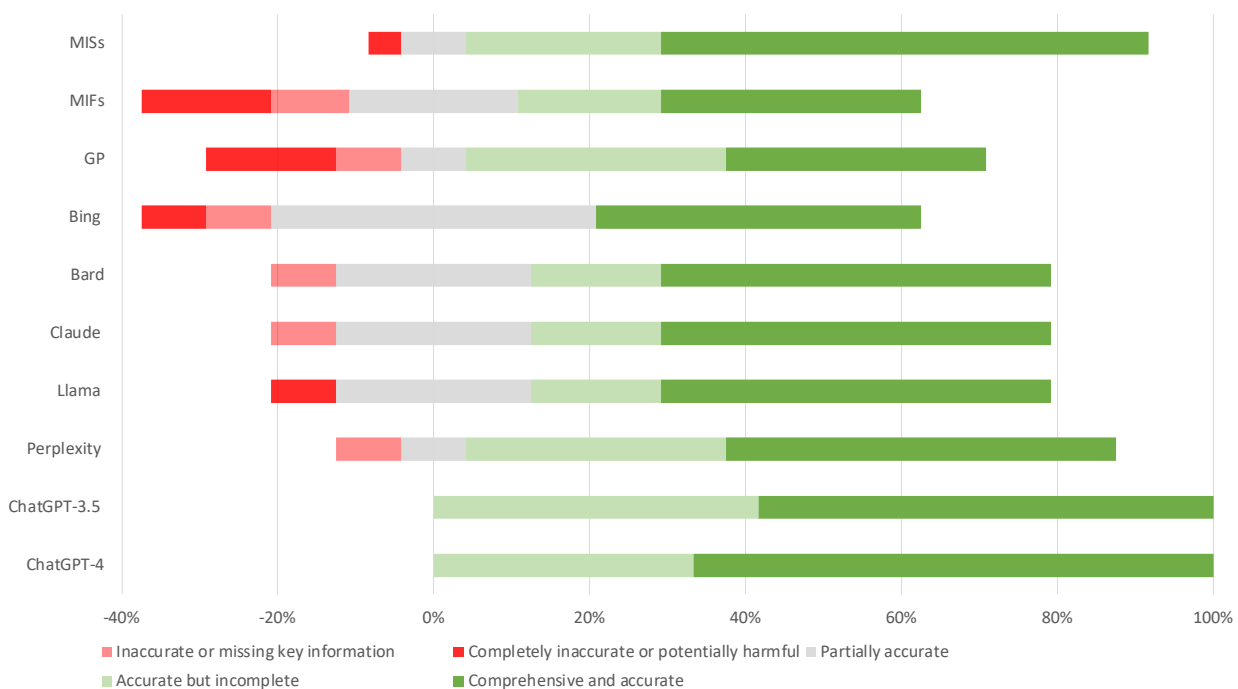

Supplement: Supplementary file 1 — Supporting Information Additional supporting information can be found online in the Supporting Information section. [file JOBE-2026-2376530-s001.zip › Fig_A9.pdf]
